# Changing Incidence, Aetiology and Outcomes of Prosthetic Joint Infections: A Population-Based Study in Iceland

**DOI:** 10.3390/jcm14155289

**Published:** 2025-07-26

**Authors:** Ingunn Haraldsdóttir, Signy Lea Gunnlaugsdóttir, Dagur Fridrik Kristjánsson, Helga Erlendsdóttir, Kristján Orri Helgason, Elías Þór Gudbrandsson, Bryndís Sigurdardóttir, Magnús Gottfredsson

**Affiliations:** 1 Faculty of Medicine, School of Health Sciences, University of Iceland, 101 Reykjavik, Iceland; inh30@hi.is (I.H.); dagurf@landspitali.is (D.F.K.); 2 Department of Medicine, Landspitali University Hospital, 105 Reykjavik, Iceland; 3 Department of Clinical Microbiology, Landspitali University Hospital, 101 Reykjavik, Iceland; 4 Department of Orthopaedic Surgery, Landspitali University Hospital, 105 Reykjavik, Iceland; 5 Department of Infectious Diseases, Landspitali University Hospital, 105 Reykjavik, Iceland; 6 Department of Science, Landspitali University Hospital, 105 Reykjavik, Iceland

**Keywords:** prosthesis-related infections, epidemiology, incidence, arthroplasty, microbiology, debridement, treatment outcome

## Abstract

**Background/Objectives**: The rising demand for total joint arthroplasty (TJA) and increasing incidence of prosthetic joint infections (PJIs) significantly burden patients and healthcare systems. This retrospective study describes the epidemiology, clinical characteristics and outcomes of PJIs in Iceland from 2003 to 2020. **Methods**: PJI cases were identified through synovial fluid cultures and ICD codes, with classification per EBJIS criteria. Unlikely cases were excluded. **Results**: Among 293 cases with a mean age of 70 years, 60% (176/293) were males and 58% (171/293) involved the knee. Over half of infections occurred within two years post TJA, with an incidence rate of 0.94%, increasing significantly over time (*p* = 0.012). Males had significantly higher incidence rates than females (incidence rate ratio 0.42; *p* < 0.001). The most common pathogens were coagulase-negative staphylococci (30%, 88/293), and 9% (27/293) of cases were culture-negative. DAIR was the first-line treatment in about 50% (147/293) of cases but it failed in nearly half, contributing to an overall treatment failure rate of 38% (98/259). PJI-related mortality was 2% (6/293). **Conclusions**: The results indicate an increased incidence, with the highest risk within two years of TJA. Males are at greater risk, while females more commonly undergo TJA. DAIR success rates were lower than reported elsewhere but improved significantly over time. Better methods to prevent PJIs are needed.

## 1. Introduction

The demand for total joint arthroplasty (TJA) has increased significantly over the last few decades and is expected to continue [1]. Therefore, the cumulative burden of prosthetic joint infections (PJIs) is increasing. The accurate diagnosis of PJIs poses a significant challenge as there is now a greater understanding of low-grade infections that may have been missed in the past, partly due to high rates of false-negative tests associated with low-virulent organisms and culture-negative cases. This has called for an increased sensitivity of diagnostic methods and markers [2], with currently no test able to definitively exclude PJIs [3,4]. Previous studies have used a variety of diagnostic criteria, making comparison difficult. The European Bone and Joint Infection Society (EBJIS) recently proposed a three-level approach to the diagnosis, which ultimately yields three categories: unlikely infection, likely infection and confirmed infection. The EBJIS definition has been shown to increase sensitivity, not at the expense of specificity, by diagnosing more cases of culture-negative infections, with up to 28% of cases having no confirmatory microbiological culture [5,6].

To effectively cure a PJI, a surgical approach in combination with antibiotics is required in most cases. First-line surgical procedures primarily consist of debridement, antibiotics and implant retention (DAIR); other approaches include one-stage and two-stage prosthesis exchange procedures. Arthrodesis (fusion), device removal without reimplantation (Girdlestone procedure) and amputation are used as treatments of last resort or in rescue treatment in refractory PJIs.

DAIR procedures, although associated with lower morbidity, are generally less successful than exchange procedures [7,8], with success rates varying widely and failure rates as high as 80% having been reported [9,10]. More recent meta-analyses estimate a success rate of 60–67%, with an improved selection of patients considered eligible for implant retention [11]. Risk factors for DAIR failure include delayed diagnosis, presence of a sinus tract, older age, surgical factors such as the inability to exchange removable prosthetic components and certain microbial factors [12,13,14,15]. For patients who are not considered surgical candidates, long-term or lifelong antibiotic suppressive therapy may be the only option.

Iceland is exceptionally well-suited for population-based studies due to the high quality of retrievable clinical data and universal coverage of healthcare, but only two limited studies are available on PJIs. One looked at outcomes after knee arthroplasty at a small district hospital and the other focused on a private clinic, with both showing infection rates of 0.6–0.7% [16,17].

This nationwide study aims to describe the epidemiology, clinical characteristics, microbiology and treatment approaches in PJIs and their associations with outcomes in Iceland over 18 years, from 2003 to 2020.

## 2. Materials and Methods

### 2.1. Study Design, Sources of Data and Ethics Approval

This retrospective study included all known patients with hip, knee or shoulder PJIs in Iceland. Two approaches were used to identify patients: First, a nationwide computerised and manual search for positive synovial fluid cultures was conducted in microbiology departments. Second, a search for ICD codes (T84.5, M00) for patients with a discharge diagnosis of PJI was performed, covering an 18-year period, from 1 January 2003 to 31 December 2020. Primary and revision arthroplasty procedure data were acquired from the Directorate of Health and used as the numerator in incidence calculations (cases/procedures). Due to incomplete data before 2010, we used the number of knee and hip joint arthroplasties performed over 11 years (2010–2020) to calculate incidence per procedure. Additionally, information on the number of overseas arthroplasties covered by Icelandic Health Insurance was obtained. The study was approved by the National Bioethics Committee (15-008-V3, 22 March 2022).

### 2.2. Data Collection and Case Definitions

Medical records of all identified patients were reviewed for the following data: age at the time of diagnosis, sex, comorbidities, joint involved, date of index joint surgery, clinical presentation, laboratory test results, microbiology results from joint fluid and intra-operative samples and surgical management. Patients were categorised into three groups: confirmed infection, likely infection and unlikely infection based on EBJIS guidance [5]. Patients with an unlikely infection were excluded.

Zimmerli classification was used to classify PJIs based on the time to infection: early-onset infections occur <3 months after the index arthroplasty, delayed-onset infections occur 3–24 months after the procedure and late-onset infections occur >24 months after arthroplasty [18]. A PJI is generally considered more likely to be related to the joint replacement surgery if the infections have an onset < 24 months after index surgery.

We also looked at presentation type as defined in the recent literature: early postoperative PJIs diagnosed ≤ 30 days after index surgery, late-acute PJIs diagnosed > 30 days after index surgery but with ≤7 days of symptoms and chronic PJIs diagnosed > 30 days after index surgery and >30 days of symptoms. The remainder were considered not classifiable [19].

Treatment failure was defined as (a) a need for any further surgical procedure related to infection, (b) use of long-term suppressive antibiotics and (c) PJI-related death, defined as in-hospital death or death occurring within 30 days of a PJI diagnosis. The minimum follow-up time for patients who developed a PJI was two years after diagnosis, with follow-up continuing until either death or the end of 2022, whichever occurred first.

### 2.3. Epidemiology

Incidence per procedure was calculated as the number of confirmed and likely hip and knee infections diagnosed within the first 24 months from the index procedure (i.e., early and delayed infections), using the number of knee and hip joint arthroplasties performed over an 11-year period (2010–2020) as the denominator. To account for varying follow-up time, person-years were used for calculations of PJI incidence per procedure. Follow-up time for cases was directly calculated, while for infection-free procedures, a follow-up time of two years was estimated. Sex- and age-specific incidence rates were also calculated for the period from 2003 to 2020 by dividing the number of early and delayed infections, including shoulder infections, by the sex- and age-specific Icelandic population as listed by Statistics Iceland (cases/100,000 individuals > 18 years/year).

### 2.4. Patient Involvement

There was no active patient or public involvement.

### 2.5. Statistical Analysis

Statistical analysis was performed using R v4.1.3 (R Core Team, Vienna, Austria). The χ^2^ test and Fisher’s exact test were used for categorical variables. Normality was assessed using the Shapiro–Wilk test and visual inspection of histograms and Q-Q plots. No major deviations from normality were observed. Average differences were analysed using the *t*-test, while median differences were evaluated using the Kruskal–Wallis test and Wilcoxon test. Because model diagnostics indicated that a Poisson regression was not adequate, negative binomial regression models were used to assess the incidence rate effects by the independent variables age, gender, infected joint and year. Models with two-way and three-way interactions showed no significant interactions. A logistic regression evaluated the effect of several independent variables, namely treatment (a categorical variable with three main treatment options: antibiotics without curative surgery, two-stage exchange and DAIR as a reference group), age, gender and primary versus revision surgeries, on treatment status (success/failure) as an outcome. Model fit was verified using the Hosmer–Lemeshow goodness-of-fit test. Two-tailed testing was performed, and *p* < 0.05 was used as the level of significance.

## 3. Results

### 3.1. Identification of PJIs

**Figure 1:**
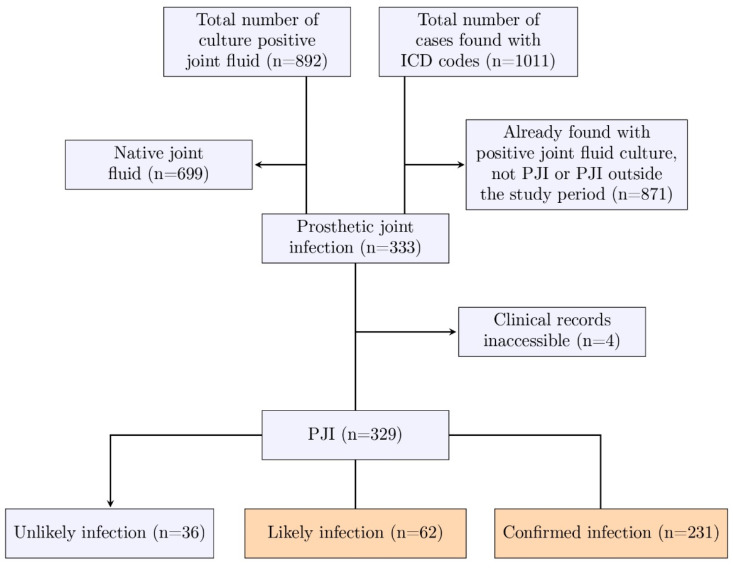
Flow chart showing identification of PJI cases, 2003–2020.

A flow diagram showing how cases were identified is shown in Figure 1. The final cohort consisted of 293 patients with a confirmed or likely diagnosis of PJI.

### 3.2. Demographics and Clinical Characteristics

Table 1 shows patient demographics by onset of PJI (Zimmerli classification). Overall, 60% (176/293) were male and 40% (117/293) were female (*p* < 0.001), with a mean age of 69.6 years. The infections were early or delayed PJIs (diagnosed within 2 years from arthroplasty) in 54% (157/293) of cases, with knee prosthetic infections being most common (58%, 171/293). Table S1 shows laboratory results at diagnosis.

### 3.3. Incidence

**Figure 2:**
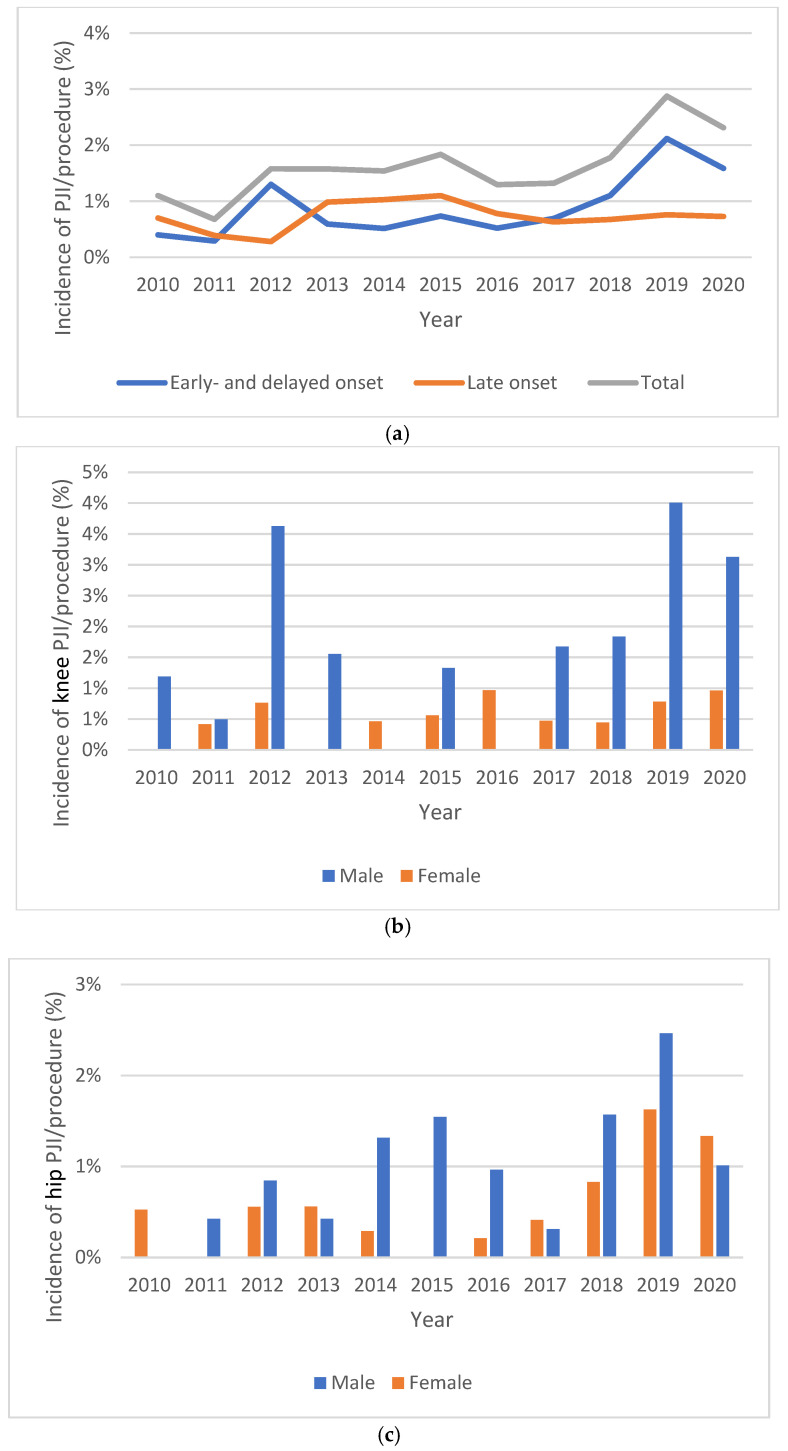
(**a**) Incidence rate for all hip and knee PJIs from 2010 to 2020. Early and delayed infections (PJI < 2 years after index surgery), late infections (>2 years after index surgery) and all PJIs combined. (**b**) Early and delayed infection rates per knee procedure from 2010 to 2020. (**c**) Early and delayed infection rates per hip procedure from 2010 to 2020.

The total number of knee and hip arthroplasties performed between 2010 and 2020 was 13,528. Overall, 57% (7771/13,528) of arthroplasty patients were female and 43% (5757/13,528) were male. Figure 2a shows the incidence rate for all hip and knee PJIs from 2010 to 2020 for infections diagnosed < 2 years after index surgery (early- and delayed-onset PJIs), >2 years after index surgery (late-onset PJI) and all PJIs combined. The average incidence of PJIs was 1.66% overall; it was 2.38% for males and 1.11% for females.

Table 2 shows adjusted incidence rate ratios (IRRs) for PJIs across different variables. For early and delayed infections only, the average incidence was 0.94% (1.41% in males and 0.59% in females (IRR = 0.42, *p* < 0.001)), with a significant increase from 2016 to 2020 compared to 2010–2015 (IRR = 1.82, *p* = 0.012).

Figure 2 shows the incidence rates of early and delayed infections per knee (Figure 2b) and hip (Figure 2c) procedure among males and females. From 2010 to 2020, the overall incidence rate for early and delayed knee infections was 1.48% (1.84% in males and 0.59% in females (IRR = 0.32, *p* < 0.001)), with an upward trend that was not statistically significant (IRR = 1.64, *p* = 0.231). For the same period, the incidence rate for early and delayed hip infections was 0.84% (0.97% in males and 0.60% in females (IRR = 0.61, *p* = 0.119)), with a significant increase in the incidence rate seen when comparing 2016–2020 to 2010–2015 (IRR = 2.05, *p* = 0.025).

### 3.4. Microbial Aetiology

Table 3 details the pathogens isolated from joint fluid, intra-operative tissue samples or both, with 63% (186/293) of isolates being identified in joint fluid. Tissue sampling during surgery yielded an additional 27% (80/293) of pathogens.

Coagulase-negative staphylococci (CoNS) were most common, found in 30% (88/293) of cases, where *Staphylococcus epidermidis* was most common, followed by *Staphylococcus lugdunensis*. CoNS infections were more frequently associated with early- and delayed-onset PJIs compared to late-onset PJIs (*p* = 0.004). *Staphylococcus aureus* (*S. aureus*) was the second most common pathogen, identified in 26% (76/293) of cases. There was no significant difference seen in pathogens considering gender or joint type. When microbiological findings were analysed in the context of presentation type, CoNS and *Cutibacterium acnes* were significantly associated with late-onset and a more protracted course, whereas *Staphylococcus aureus* was associated with acute presentations (Table 4). Culture-negative (CN) PJIs in this study accounted for 9% (27/293) of cases; see Table S2 for comparison with culture-positive (CP) PJIs.

### 3.5. Treatment and Outcome

In total, 80% (234/293) of infections were initially treated with DAIR, two-stage exchange or antibiotics without curative surgery. Table 5 compares patients by initial treatment approach.

DAIR was used in roughly 50% (147/293) of cases, predominantly for early-onset infections, while two-stage exchange was chosen in 20% (60/293) of cases, mainly for late-onset infections. A logistic regression model was employed to examine the relationship between treatment success as an outcome and several independent variables: the three main treatment options, age, gender and primary versus revision surgeries. The model revealed significantly higher odds of treatment success in the two-stage exchange group than the DAIR (reference) group (OR = 7.70; *p* < 0.001), while the other variables did not yield significant results.

Table 6 compares outcomes of initial treatments, excluding patients who received antibiotics as a first-line treatment without curative intent. Treatment failure occurred in 38% (98/259) of cases, with PJI mortality at 2.3% (6/259). A PJI was considered a contributory cause of death according to death certificates in a further 7.3% (19/259) of cases, all occurring within two years from diagnosis.

DAIR successes and failures are compared in Table S3. During the first half of the study period, there was a 62% failure rate following DAIR approach compared to a 42% failure rate in the second half (2003–2011: 21/34 vs. 2012–2020: 47/113; *p* = 0.050).

## 4. Discussion

This nationwide study provides a epidemiological, clinical and prognostic analysis of patients with PJIs over 18 years. We found that the incidence of PJIs per procedure significantly increased during the study period. The average incidence rate for knee and hip PJIs, considered related to the index surgery, was 0.94%—0.84% for hip arthroplasties and 1.48% for knee arthroplasties. This is comparable to previous studies, where 1% was the estimated incidence rate for hip arthroplasties and 1–2.5% was the estimated incidence rate for knee arthroplasties, although comparison can be difficult due to variable inclusion criteria and definitions of PJIs [20,21,22,23,24]. Additionally, many previous reports on PJIs are database studies that use records of revision arthroplasty for infection as a means of estimating PJIs following arthroplasty, which has been shown to substantially underestimate the true incidence of these infections. This is because other treatment options can be non-surgical, most commonly antibiotic treatment, or involve other surgical procedures such as debridement, joint fusion or amputation [24,25]. Validity studies of joint registries have shown low sensitivity in capturing infection prevalence; however, sensitivity can be markedly increased by combining data from microbiology databases [25,26]. Finally, we included PJIs following both primary and revision arthroplasties, whilst some studies only include infections following primary arthroplasty. Infection rate is higher after revision surgery [27], and thus our real-world nationwide data may provide a more accurate estimate. The variability in incidence estimates from different studies underlines the importance of good documentation and the standardisation of diagnostic criteria of these infections.

The reason for the increasing incidence of PJIs is uncertain but is likely to be multifactorial. It is possible that with an ageing population and longer prosthesis lifetime, there are more high-risk revision procedures resulting in a higher infection rate; however, no significant increase in the mean age of patients with infection was observed during the study period. There was an increase in PJIs after revision surgery during the study period, but unfortunately, we did not possess information on the total number of revisions compared to primary arthroplasties to calculate the rates. In addition to surgical and demographic factors, the pathogenesis of PJIs involves a sustained inflammatory process, triggered by bacterial biofilm formation. As described by Moldovan [28], systemic biomarkers are significantly elevated in PJIs, supporting their value in improving diagnostic accuracy and understanding the inflammatory burden in affected patients.

### 4.1. Clinical Characteristics

Early- and delayed-onset PJIs accounted for 54% of all PJI cases, which is in line with previous studies showing that the risk of a PJI is greatest within two years of the insertion of an artificial joint [23]. Gender differences are noteworthy, with males being significantly more likely to develop PJIs, whilst more women underwent arthroplasties every year from 2010 to 2020. The adjusted incidence rate ratio indicates that the infection rate among women was approximately 58% lower than among men. This male predominance, also seen in native joint infections, is in line with earlier reports, but the reasons for this difference remain uncertain [22,23,24,29,30,31,32].

### 4.2. Diagnoses and Microbiology

It is noteworthy that only 63% of isolated bacteria were found with joint fluid sampling, underlining the importance of intra-operative sampling to increase diagnostic yield. According to our study, CoNS were the most common causes, whereas most previous studies report *S. aureus* to be most common [20,32,33]. We can only speculate what the reasons may be. Historically, methicillin-resistant *Staphylococcus aureus* (MRSA) has been very uncommon in the hospital environment in Iceland, and it was not identified in a single case of PJI, whereas in other cohorts, MRSA has played a very substantial role [20,31,32]. According to our results, patients with early and delayed PJIs were significantly more likely to have CoNS and polymicrobial infections when compared with late-onset PJIs, suggesting it is directly related to the arthroplasty. Similarly, when the microbial aetiology was analysed by presentation type, as proposed by Davis et al., *S. aureus* was more likely to be associated with early postoperative and late-acute presentations, whereas *C. acnes* was significantly associated with more chronic presentation. The same authors recently reported that the lowest treatment success rates in the management of PJIs (46%) involved both of these pathogens as well as Gram-negative rods [19].

A total of 9% of PJIs in our study were classified as culture-negative (CN), similar to previous reports [21,34,35]. CN PJIs may be underdiagnosed in our study since not all tests that make up the criteria were performed on our patients [5]. Tests such as alpha-defensin and synovial fluid cell counts were only occasionally performed, and sonication and high-power field histology were not routinely performed.

### 4.3. Treatment Failure

When excluding patients who were initially treated with long-term antimicrobial suppression, overall treatment failure occurred in 38% of PJIs. CN PJIs were less likely to have treatment failure compared to CP PJIs. The success from DAIR increased during the study period, from 38% to 58%, which is close to the success rates described in recent meta-analyses [7,8,11]. This likely reflects an improved selection of patients as well as the further optimisation of intra- and postoperative management. Factors such as early intervention, avoidance of high-risk patients and modular component exchange have been shown to increase success [36]. As outlined in the EBJIS position paper, DAIR is contraindicated in patients with loose implants, prolonged symptom duration exceeding three weeks or the presence of a sinus tract. However, it may still be considered as a palliative or rescue strategy in medically unfit patients.

### 4.4. Limitations

This study has several limitations. In data collection, coding accuracy was lower in the earlier years of the study period, which may have led to underreporting or a misclassification of infections during that time. Another notable limitation of this study is the absence of a standardised diagnostic protocol. The use of different criteria across sites and over time might affect case ascertainment. In addition, we did not adjust for potential confounders such as diabetes mellitus, obesity and revision status in the multivariate models.

## 5. Conclusions

The incidence of PJIs in Iceland is increasing according to this long-term nationwide study. Men are more likely to have PJIs following arthroplasty compared to women, but more women undergo arthroplasties. Less than two-thirds of isolated bacteria were found by joint fluid sampling, which underlines the importance of intra-operative tissue sampling to increase diagnostic sensitivity and accuracy. DAIR success rates were low compared to previous studies but improved significantly during the second half of the study period, most likely due to an improved selection of surgical candidates. Even when the risk of failure with DAIR is high, it may still be reasonable to achieve source control when alternative surgical strategies are unacceptable, when cure is not expected and the patient receives long-term antibiotic suppression following debridement. Our study suggests that better methods to prevent PJIs are needed. These may include, but are not limited to, prehabilitation, the optimisation of antimicrobial prophylaxis and methods to reduce biofilm formation on prosthetic material.

## Figures and Tables

**Table 1:** Patient demographics by onset of PJI (Zimmerli classification).

	Early Onset ^a^ (*n* = 92)	Delayed Onset ^a^ (*n* = 65)	Late Onset ^a^ (*n* = 136)	Overall (*n* = 293)	*p*-Value ^b^
Gender					
Male	55 (59.8%)	43 (66.2%)	78 (57.4%)	176 (60.1%)	0.445
Female	37 (40.2%)	22 (33.8%)	58 (42.6%)	117 (39.9%)	
EBJIS criteria					
Likely PJI	19 (20.7%)	14 (21.5%)	29 (21.3%)	62 (21.2%)	1.000
Confirmed PJI	73 (79.3%)	51 (78.5%)	107 (78.7%)	231 (78.8%)	
Age (years)					
Median [IQR]	69.1 [64.5–76.4]	68.7 [62.9–75.9]	71.0 [64.6–79.5]	69.3 [64.3–77.4]	0.165
Primary vs. Revision arthroplasty					
Primary	66 (71.7%)	42 (64.6%)	70 (51.5%)	178 (60.8%)	0.436
Revision	19 (20.7%)	15 (23.1%)	16 (11.8%)	50 (17.1%)	
Missing	7 (7.6%)	8 (12.3%)	50 (36.8%)	65 (22.2%)	
Presentation type ^c^					
Early postoperative	45 (48.9%)	0 (0%)	0 (0%)	45 (15.4%)	**<0.001**
Late acute	28 (30.4%)	39 (60.0%)	89 (65.4%)	156 (53.2%)	
Chronic	6 (6.5%)	16 (24.6%)	17 (12.5%)	39 (13.3%)	
Late (Not classifiable)	12 (13.0%)	9 (13.8%)	24 (17.6%)	45 (15.4%)	
Missing	1 (1.1%)	1 (1.5%)	6 (4.4%)	8 (2.7%)	
Duration of symptoms (days)					
Median [IQR]	5 [2–14]	5 [2–33.5]	3 [1–14]	4.00 [1–14]	0.147
Infected joint					
Knee	46 (50.0%)	47 (72.3%)	78 (57.4%)	171 (58.4%)	0.836
Hip	44 (47.8%)	17 (26.2%)	55 (40.4%)	116 (39.6%)	0.875
Shoulder	2 (2.2%)	1 (1.5%)	3 (2.2%)	6 (2.0%)	1.000
Symptoms at admission to hospital (% of available data) ^d^					
Joint pain	68/92 (73.9%)	63/65 (96.9%)	128/136 (94.1%)	259/293 (88.4%)	**0.008**
Swollen joint	55/78 (70.5%)	44/63 (69.8%)	74/127 (58.3%)	173/268 (64.6%)	0.093
Warm joint	36/75 (48.0%)	33/62 (53.2%)	53/123 (43.1%)	122/260 (46.9%)	0.347
Joint redness	50/81 (61.7%)	22/62 (35.5%)	32/125 (25.6%)	104/268 (38.8%)	**<0.001**

^a^ Early-onset infections occurring <3 months after TJA, delayed-onset infections occurring 3–24 months after TJA, late-onset infections occurring >24 months after TJA (Zimmerli classification). ^b^ *p*-value calculated for early- and delayed-onset PJIs vs. late-onset PJIs, with values < 0.05 shown in bold. ^c^ Presentation type defined as follows: Early postoperative PJIs diagnosed ≤ 30 days after index surgery, late-acute PJIs diagnosed >30 days after index surgery with ≤7 days of symptoms, chronic PJIs diagnosed > 30 days after index surgery and >30 days of symptoms [19]. ^d^ Symptoms present on admission; the denominator indicates available data regarding symptoms.

**Table 2:** Negative binomial regression results showing the adjusted incidence rate ratios (IRRs) for PJI incidence across different variables.

Variables	IRR Adjusted	Confidence Interval	*p*-Value
PJI/procedure-person-years ^a^			
Gender (female vs. male)	0.42	[0.27–0.68]	**<0.001**
Joint (knee vs. hip infection)	1.37	[0.90–2.22]	0.169
Period (2016–2020 vs. 2010–2015)	1.82	[1.11–2.82]	**0.012**
PJI/general population-person-years ^b^			
Gender (female vs. male)	0.63	[0.42–0.93]	**0.022**
Period (2012–2020 vs. 2003–2011)	3.01	[1.94–4.65]	**<0.001**

^a^ Incidence rate ratios for early and delayed knee and hip infection per person-year at risk due to procedure, for the period 2010–2020. ^b^ Incidence rate ratios for early and delayed knee, hip and shoulder infection in person-years based on yearly census data of the general population aged ≥ 18 years, for the period 2003–2020. *p*-values < 0.05 are shown in bold.

**Table 3:** Microbiology of PJIs in Iceland 2003–2020 by onset of PJI (Zimmerli classification).

Pathogen Isolate	Early Onset ^a^ (*n* = 92)	Delayed Onset ^a^ (*n* = 65)	Late Onset ^a^ (*n* = 136)	Total (*n* = 293)	*p*-Value ^b^
Coagulase-negative staphylococci ^c^	39 (42.4%)	19 (29.2%)	30 (22.1%)	88 (30.0%)	**0.004**
*Staphylococcus aureus* ^d^	24 (26.1%)	13 (20.0%)	39 (28.7%)	76 (25.9%)	0.259
Streptococci ^e^	4 (4.3%)	16 (24.6%)	29 (21.3%)	49 (16.7%)	0.071
Enterococci	7 (7.6%)	1 (1.5%)	4 (2.9%)	12 (4.1%)	0.527
*Cutibacterium acnes*	1 (1.1%)	4 (6.2%)	3 (2.2%)	8 (2.7%)	0.729
Candida species	2 (2.2%)	1 (1.5%)	3 (2.2%)	6 (2.0%)	1.000
Clostridium species	3 (3.3%)	0 (0%)	1 (0.7%)	4 (1.4%)	0.220
Corynebacterium species	2 (2.2%)	0 (0%)	2 (1.5%)	4 (1.4%)	1.000
Gram-negative bacteria	5 (5.4%)	0 (0%)	5 (3.7%)	10 (3.4%)	0.738
*Escherichia coli*	1 (1.1%)	0 (0%)	4 (2.9%)	5 (1.7%)	
*Proteus mirabilis*	2 (2.2%)	0 (0%)	1 (0.7%)	3 (1.0%)	
*Klebsiella pneumoniae*	1 (1.1%)	0 (0%)	0 (0%)	1 (0.3%)	
*Pseudomonas aeruginosa*	1 (1.1%)	0 (0%)	0 (0%)	1 (0.3%)	
Other pathogen	2 (2.2%)	3 (4.6%)	4 (2.9%)	9 (3.1%)	0.756
Polymicrobial ^f^	22 (23.9%)	7 (10.8%)	9 (6.6%)	38 (13.0%)	**0.003**
Culture-negative	3 (3.3%)	8 (12.3%)	16 (11.8%)	27 (9.2%)	0.229

^a^ Early-onset infections occurring <3 months after TJA, delayed-onset infections occurring 3–24 months after TJA, late-onset infections occurring >24 months after TJA. ^b^ *p*-value calculated for early- and delayed-onset PJIs vs. late-onset PJIs with values < 0.05 shown in bold. ^c^ The most common species were *Staphylococcus epidermidis* and *Staphylococcus lugdunensis*. ^d^ All cases were caused by methicillin-susceptible *Staphylococcus aureus*. ^e^ Beta-hemolytic streptococci in 29 cases, viridians group streptococci in 13 cases. ^f^ Polymicrobial PJI: In 16/38 cases, CoNS was thought to be the main causative organism; in 6/38 cases, it was *S. aureus*; and in 4/38 cases, different streptococci were the main pathogens.

**Table 4:** Microbiology according to presentation type ^a^.

Pathogen Isolate	Early Postoperative (*n* = 45)	Late Acute (*n* = 156)	Chronic (*n* = 39)	Late (Not Classifiable) (*n* = 45)	Total (*n* = 285) ^b^	*p*-Value ^c^
Coagulase-negative staphylococci ^d^	15 (33.3%)	33 (21.2%)	19 (48.7%)	20 (44.4%)	87 (30.5%)	**<0.001**
*Staphylococcus aureus*	12 (26.7%)	52 (33.3%)	4 (10.3%)	6 (13.3%)	74 (26.0%)	**<0.001**
Streptococci ^e^	1 (2.2%)	36 (23.1%)	2 (5.1%)	10 (22.2%)	49 (17.2%)	0.504
Enterococci	6 (13.3%)	5 (3.2%)	0 (0%)	1 (2.2%)	12 (4.2%)	0.119
*Cutibacterium acnes*	0 (0%)	2 (1.3%)	6 (15.4%)	0 (0%)	8 (2.8%)	**0.009**
*Candida* species	1 (2.2%)	2 (1.3%)	1 (2.6%)	1 (2.2%)	5 (1.8%)	0.634
*Clostridium* species	1 (2.2%)	2 (1.3%)	0 (0%)	1 (2.2%)	4 (1.4%)	1.000
*Corynebacterium* species	1 (2.2%)	1 (0.6%)	0 (0%)	2 (4.4%)	4 (1.4%)	0.827
Gram-negative bacteria	5 (11.1%)	4 (2.6%)	0 (0%)	0 (0%)	9 (3.2%)	0.110
*Escherichia coli*	1 (2.2%)	3 (1.9%)	0 (0%)	0 (0%)	4 (1.4%)	
*Proteus mirabilis*	2 (4.4%)	1 (0.6%)	0 (0%)	0 (0%)	3 (1.1%)	
*Klebsiella pneumoniae*	1 (2.2%)	0 (0%)	0 (0%)	0 (0%)	1 (0.4%)	
*Pseudomonas aeruginosa*	1 (2.2%)	0 (0%)	0 (0%)	0 (0%)	1 (0.4%)	
Other pathogen	2 (4.4%)	3 (1.9%)	1 (2.6%)	3 (6.7%)	9 (3.2%)	0.488
Polymicrobial ^f^	17 (37.8%)	13 (8.3%)	5 (12.8%)	3 (6.7%)	38 (13.3%)	0.302
Culture-negative	1 (2.2%)	*16 (10.3%)*	*6 (15.4%)*	1 (2.2%)	*24 (8.4%)*	1.000

^a^ Presentation type defined as follows: Early postoperative PJIs diagnosed ≤ 30 days after index surgery, late-acute PJIs diagnosed >30 days after index surgery with ≤7 days of symptoms, chronic PJIs diagnosed > 30 days after index surgery and >30 days of symptoms [19]. ^b^ Only 285 of 293 cases had available data on presentation type. ^c^ *p*-value calculated for early postoperative and late acute PJIs vs. chronic and other PJIs with values < 0.05 shown in bold. ^d^ The most common species were *Staphylococcus epidermidis* and *Staphylococcus lugdunensis*. ^e^ Beta-hemolytic streptococci were most common. ^f^ Polymicrobial PJI: In 16/38 cases, CoNS was thought to be the main causative organism; in 6/38 cases, it was *S. aureus*; and in 4/38 cases, different streptococci were the main pathogens.

**Table 5:** Most common initial treatment approaches ^a^.

	DAIR (*n* = 147)	Antibiotics without Curative Surgery ^b^ (*n* = 27)	Two-Stage (*n* = 60)	Overall (*n* = 234)	*p*-Value
Gender					
Male	93 (63.3%)	16 (59.3%)	33 (55.0%)	142 (60.7%)	0.536
Female	54 (36.7%)	11 (40.7%)	27 (45.0%)	92 (39.3%)	
Age (years)					
Median [IQR]	69.7 [64.1–77.4]	69.7 [63.3–74.1]	67.2 [62.0–73.6]	68.8 [63.6–76.2]	0.150
Infected joint, *n* (%)					
Knee	89 (60.5%)	18 (66.7%)	36 (60.0%)	143 (61.1%)	0.818
Hip	55 (37.4%)	8 (29.6%)	24 (40.0%)	87 (37.2%)	0.648
Shoulder	3 (2.0%)	1 (3.7%)	0 (0%)	4 (1.7%)	0.411
Zimmerli classification					
Early onset	77 (52.4%)	5 (18.5%)	8 (13.3%)	90 (38.5%)	**<0.001**
Delayed onset	26 (17.7%)	9 (33.3%)	14 (23.3%)	49 (20.9%)	0.161
Late onset	44 (29.9%)	13 (48.1%)	38 (63.3%)	95 (40.6%)	**<0.001**
Duration of symptoms (days)					
Median [IQR]	4 [2–13.5]	2 [1–10.5]	7 [1–95]	4 [1–14]	**0.021**
Missing	5 (3.4%)	0 (0%)	1 (1.7%)	6 (2.6%)	
Pathogen isolate, *n* (%)					
Coagulase-negative staphylococci ^c^	45 (30.6%)	8 (29.6%)	22 (36.7%)	75 (32.1%)	0.671
*Staphylococcus aureus*	42 (28.6%)	7 (25.9%)	8 (13.3%)	57 (24.4%)	**0.045**
Streptococci ^d^	27 (18.4%)	4 (14.8%)	8 (13.3%)	39 (16.7%)	0.704
Enterococci	7 (4.8%)	0 (0%)	2 (3.3%)	9 (3.8%)	0.779
*Cutibacterium acnes*	0 (0%)	1 (3.7%)	5 (8.3%)	6 (2.6%)	**0.002**
*Candida* species	3 (2.0%)	0 (0%)	0 (0%)	3 (1.3%)	0.695
*Clostridium* species	3 (2.0%)	0 (0%)	1 (1.7%)	4 (1.7%)	-
*Corynebacterium* species	3 (2.0%)	0 (0%)	1 (1.7%)	4 (1.7%)	1.000
Gram-negative bacteria	5 (3.4%)	0 (0%)	2 (3.3%)	7 (3.0%)	1.000
*Escherichia coli*	2 (1.4%)	0 (0%)	1 (1.7%)	3 (1.3%)	-
*Proteus mirabilis*	1 (0.7%)	0 (0%)	1 (1.7%)	2 (0.9%)	-
*Klebsiella pneumoniae*	1 (0.7%)	0 (0%)	0 (0%)	1 (0.4%)	-
*Pseudomonas aeruginosa*	1 (0.7%)	0 (0%)	0 (0%)	1 (0.4%)	
Other	4 (2.7%)	2 (7.4%)	3 (5.0%)	9 (3.8%)	0.440
Polymicrobial	26 (17.7%)	3 (11.1%)	7 (11.7%)	36 (15.4%)	0.529
Culture-negative	8 (5.4%)	5 (18.5%)	8 (13.3%)	21 (9.0%)	**0.026**

^a^ Other initial treatments not included in this table (*n* = 58) are lifelong suppressive antibiotic therapy, one-stage, Girdlestone, arthrodesis and amputation. One patient died before receiving initial treatment. ^b^ Curative surgery: part of a recognised management option for PJIs with a curative intent. ^c^ The most common species were *Staphylococcus epidermidis* and *Staphylococcus lugdunensis*. ^d^ Beta-hemolytic streptococci were most common. *p*-values < 0.05 are shown in bold.

**Table 6:** Comparison of treatment failure and treatment success.

	Treatment Success (*n* = 161)	Treatment Failure (*n* = 98)	Overall (*n* = 259) ^a^	*p*-Value ^b^
Gender				
Male	98 (60.9%)	58 (59.2%)	156 (60.2%)	0.968
Female	63 (39.1%)	40 (40.8%)	103 (39.8%)	
Age (years)				
Median [IQR]	68.8 [64.6–75.5]	68.8 [62.9–78.0]	68.8 [64.1–76.2]	0.914
Joint affected				
Knee	96 (59.6%)	53 (54.1%)	149 (57.5%)	0.512
Hip	59 (36.6%)	45 (45.9%)	104 (40.2%)	0.210
Shoulder	6 (3.7%)	0 (0%)	6 (2.3%)	0.086
Zimmerli classification				
Early onset	53 (32.9%)	37 (37.8%)	90 (34.7%)	0.897
Delayed onset	39 (24.2%)	20 (20.4%)	59 (22.8%)	
Late onset	69 (42.9%)	41 (41.8%)	110 (42.5%)	
Duration of symptoms (days)				
Median [IQR]	4 [1–17.8]	5 [1–14]	5 [1–16]	0.871
Missing	3 (1.9%)	4 (4.1%)	7 (2.7%)	
Pathogen isolate				
Coagulase-negative staphylococci ^c^	49 (30.4%)	35 (35.7%)	84 (32.4%)	0.423
*Staphylococcus aureus*	33 (20.5%)	30 (30.6%)	63 (24.3%)	0.082
Streptococci ^d^	31 (19.3%)	9 (9.2%)	40 (15.4%)	**0.027**
Enterococci	5 (3.1%)	5 (5.1%)	10 (3.9%)	0.509
*Cutibacterium acnes*	7 (4.3%)	1 (1.0%)	8 (3.1%)	0.265
*Candida* species	2 (1.2%)	4 (4.1%)	6 (2.3%)	0.202
*Clostridium* species	3 (1.9%)	1 (1.0%)	4 (1.5%)	1.000
*Corynebacterium* species	0 (0%)	4 (4.1%)	4 (1.5%)	**0.038**
Gram-negative bacteria	6 (3.7%)	3 (3.1%)	9 (3.5%)	0.714
*Escherichia coli*	4 (2.5%)	0 (0%)	4 (1.5%)	
*Proteus mirabilis*	1 (0.6%)	2 (2.0%)	3 (1.2%)	
*Klebsiella pneumoniae*	1 (0.6%)	0 (0%)	1 (0.4%)	
*Pseudomonas aeruginosa*	0 (0%)	1 (1.0%)	1 (0.4%)	
Other pathogen	5 (3.1%)	3 (3.1%)	8 (3.1%)	0.732
Polymicrobial	17 (10.6%)	21 (21.4%)	38 (14.7%)	**0.036**
Culture-negative	20 (12.4%)	3 (3.1%)	23 (8.9%)	**0.028**
First treatment after diagnosis of PJI ^e^				
DAIR	79 (49.1%)	68 (69.4%)	147 (56.8%)	**0.001**
Two-stage	52 (32.3%)	7 (7.1%)	59 (22.9%)	**<0.001**
One-stage	8 (5.0%)	7 (7.1%)	15 (5.8%)	0.637
Antibiotics without curative surgery	13 (8.1%)	14 (14.3%)	27 (10.4%)	0.160
Girdlestone	6 (3.7%)	1 (1.0%)	7 (2.7%)	0.261
Arthrodesis	2 (1.2%)	0 (0%)	2 (0.8%)	0.529
Amputation	1 (0.6%)	0 (0%)	1 (0.4%)	1.000

^a^ N = 259; 33 patients receiving antibiotics as first-line treatment without curative intent were excluded from this analysis, in one case information on treatment failure was missing (patient had a two-stage exchange). ^b^ *p*-value comparing treatment success vs. treatment failure with values < 0.05 shown in bold. ^c^ The most common species were *Staphylococcus epidermidis* and *Staphylococcus lugdunensis.* ^d^ Beta-hemolytic streptococci were most common. ^e^ Treatment failure occurred due to PJI-related death in 6 cases (4 patients had DAIR procedure as first treatment, 1 patient had a one-stage exchange procedure, 1 patient died before any surgical treatment).

## Data Availability

All data relevant to the study are included in the article or uploaded as online Supplemental Information.

## Supplementary Materials

The following supporting information can be downloaded at: https://www.mdpi.com/article/10.3390/jcm14155289/s1, Table S1: Laboratory results. Table S2: Baseline characteristics of culture-negative PJIs (CN) compared with culture-positive (CP) PJIs. Table S3: DAIR failure vs. success.

## Abbreviations

The following abbreviations are used in this manuscript:

TJA	Total joint arthroplasty
PJI	Prosthetic joint infection
EBJIS	The European Bone and Joint Infection Society
DAIR	Debridement, antibiotics and implant retention
IRR	Incidence rate ratio
CoNS	Coagulase-negative staphylococcus
CN	Culture-negative
CP	Culture-positive
