# Supplementary material for: Changing Incidence, Aetiology and Outcomes of Prosthetic Joint Infections: A Population-Based Study in Iceland
Source: J Clin Med. 2025 Jul 26;14(15):5289. doi: 10.3390/jcm14155289 (PMC12347521; doi:10.3390/jcm14155289)
Supplement: Supplementary file 1 [file jcm-14-05289-s001.zip › jcm-3737843-supplementary.pdf]

Table S1. Laboratory results.

	Early Onset (n = 92)	Delayed Onset (n = 65)	Late Onset (n = 136)	Total (n = 293)	p-Value [a]
Admission data:					
Temperature (median, °C)	37.0	37.5	37.4	37.3	0.773
WCC (median, ×10⁹/L)	10.0	10.2	10.9	10.3	0.284
CRP (median, mg/L)	89	89	128	103	0.237
ESR (median, mm/h)	54	72	49	54	0.239
Synovial fluid WCC (median, ×10⁶)	35,000	48,600	63,300	54,900	0.150
Normal admission values: (% of available data)					
Temperature <37.8 °C	53/80 (66.3%)	35/60 (58.3%)	75/119 (63.0%)	163/259 (62.9%)	1.000
WCC < 10.5 × 10⁹/L	47/87 (54.0%)	36/64 (56.3%)	61/131 (46.6%)	144/282 (51.1%)	0.198
CRP < 10 mg/L	7/87 (8.0%)	4/64 (6.3%)	9/129 (7.0%)	20/280 (7.1%)	1.000
ESR < 20 mm/h	11/52 (21.2%)	8/45 (17.8%)	14/74 (18.9%)	33/171 (19.3%)	1.000
Alpha defensin: (% of performed Alpha defensin tests)					
Positive	1/2 (50.0%)	6/9 (66.7%)	9/12 (75.0%)	16/23 (69.6%)	0.667

[a] p-value calculated for early- and delayed-onset PJIs vs. late-onset PJIs.

Table S2. Baseline characteristics of culture-negative PJIs (CN) compared with culture-positive (CP) PJIs.

	CN PJI (n = 27) [a]	CP PJI (n = 266)	p-Value
**EBJIS Criteria**			
Confirmed PJI	24/27 (89)	207/266 (78)	0.223
Likely PJI	3/27 (11)	59/266 (22)	
Age in years (median [IQR])	72.4 [66.8–78.5]	69.4 [64.1–77.4]	0.225
**Gender**			
Female	15/27 (55)	102/266 (38)	0.125
Male	12/27 (45)	164/266 (62)	
**Joint affected**			
Knee	17/27 (63)	154/266 (58)	0.761
Hip	9/27 (33)	107/266 (40)	0.623
Shoulder	1/27 (4)	5/266 (2)	0.443
Duration of symptoms in days (median [IQR]) [b]	4 [1–10]	4 [1–14]	0.855
**Zimmerli classification**			
Early	3/27 (11)	89/266 (33.5)	**0.030**
Delayed	8/27 (30)	57/266 (21.5)	0.118
Late	16/27 (59)	120/266 (45)	0.210
Antibiotics prior to diagnosis	6/27 (22)	76/263 (29) [c]	0.611
**Normal admission values**			
Temperature (<37.8 °C)	16/25 (64)	147/235 (63)	1.000
WBC (<10.5 × 10⁹/L)	11/26 (42)	125/265 (47)	0.413
CRP (<10 mg/L)	1/26 (4)	30/265 (11)	0.705
ESR (<20 mm/h)	2/16 (13)	31/155 (20)	0.740
**First treatment after diagnosis of PJI** [d]			
DAIR	8/27 (30)	139/265 (52)	**0.042**
Two-stage	8/27 (30)	52/265 (20)	0.324
One-stage	0	15/265 (6)	0.384

Antibiotics without curative surgery	5/27 (19)	22/265 (8)	0.087
Prolonged suppressive antibiotic therapy	4/27 (15)	29/265 (11)	0.524
Other [e]	2/27 (7)	8/265 (3)	0.234
Treatment failure [f]	3/27 (11)	94/236 (40)	**0.003**

Data are no/N (%), *p*-value comparing CN vs. CP. [a] Culture-negative PJI had a diagnosis of PJI due to (a) a WBC synovial fluid count >3000 and PMN >80% in 12/27 cases, (b) a sinus tract in 7/27 cases, (c) a WBC synovial fluid count of >3000 only in 3/27 cases, (d) positive alpha-defensin only in 2/27 cases, (e) a sinus tract and a positive alpha-defensin in 1/27 cases, (f) positive alpha defensin, WBC synovial fluid count >3000 and PMN > 80% in 1/27 cases, g) CRP > 10 mg/L and a positive nuclear imaging in one case. [b] Information on duration of symptoms before diagnoses was available in 25/27 cases in the CN group and 261/266 in the CP group. [c] CP group: Three patients with a late-onset PJI had antibiotics prior to admission and three patients with a delayed-onset PJI. [d] One patient died before first-line treatment was decided in the CP group; N 265/266. [e] CN: One patient had arthrodesis and one patient had a Girdlestone procedure. CP: One arthrodesis, six Girdlestone procedures, one amputation. [f] Defined as the need for further surgical procedure, the need for lifelong antibiotics as second line treatment or PJI-related death. A total of three patients in the CN group who all had DAIR as first-line treatment. *p*-values <0.05 are shown in bold.

Table S3. DAIR failure vs. success.

**DAIR**	**Treatment Success (*n* = 79)**	**Treatment Failure (*n* = 68)**	**Overall (*n* = 147)**	***p*-Value [a]**
Gender				
Male	53 (67.1%)	40 (58.8%)	93 (63.3%)	0.387
Female	26 (32.9%)	28 (41.2%)	54 (36.7%)	
Age (years)				
Median [IQR]	71.1 [66.1–77.0]	67.6 [62.7–78.1]	69.7 [64.1–77.4]	0.155
Joint affected				
Knee	49 (62.0%)	40 (58.8%)	89 (60.5%)	0.821
Hip	27 (34.2%)	28 (41.2%)	55 (37.4%)	0.482
Shoulder	3 (3.8%)	0 (0%)	3 (2.0%)	0.249
Zimmerli classification				
Early onset	44 (55.7%)	33 (48.5%)	77 (52.4%)	0.438 [b]
Delayed onset	14 (17.7%)	12 (17.6%)	26 (17.7%)	
Late onset	21 (26.6%)	23 (33.8%)	44 (29.9%)	
Duration of symptoms (days)				
Median [IQR]	4 [2–12]	5 [2–14]	4 [2–13.5]	0.437
Missing	2 (2.5%)	3 (4.4%)	5 (3.4%)	
Pathogen isolate				
Coagulase-negative staphylococci [c]	20 (25.3%)	25 (36.8%)	45 (30.6%)	0.186
Staphylococcus aureus	21 (26.6%)	21 (30.9%)	42 (28.6%)	0.695
Streptococci [d]	21 (26.6%)	6 (8.8%)	27 (18.4%)	**0.011**
Enterococci	4 (5.1%)	3 (4.4%)	7 (4.8%)	1.000
Candida species	0 (0%)	3 (4.4%)	3 (2.0%)	0.097
Clostridium species	3 (3.8%)	0 (0%)	3 (2.0%)	0.249
Corynebacterium species	0 (0%)	3 (4.4%)	3 (2.0%)	0.097
Gram-negative bacteria	4 (5.1%)	1 (1.5%)	5 (3.4%)	0.124
Escherichia coli	2 (2.5%)	0 (0%)	2 (1.4%)	

Proteus mirabilis	1 (1.3%)	0 (0%)	1 (0.7%)	
Klebsiella pneumoniae	1 (1.3%)	0 (0%)	1 (0.7%)	
Pseudomonas aeruginosa	0 (0%)	1 (1.5%)	1 (0.7%)	
Other pathogen	1 (1.3%)	3 (4.4%)	4 (2.7%)	0.182
Polymicrobial	8 (10.1%)	18 (26.5%)	26 (17.7%)	**0.018**
Culture-negative	5 (6.3%)	3 (4.4%)	8 (5.4%)	0.725

[a] *p*-value comparing treatment success vs. treatment failure. [b] *p*-value calculated for early- and delayed vs. late-onset PJIs with values <0.05 shown in bold. [c] The most common subspecies were *Staphylococcus epidermidis* and *Staphylococcus lugdunensis.* [d] Beta-hemolytic streptococci most common.
